# Patterns of antidepressant use among older adults in Northern Ireland: An administrative data study.

**DOI:** 10.23889/ijpds.v9i5.1654

**Published:** 2024-09-18

**Authors:** Enya Redican, Ronald McDowell, Michael Rosato, Jamie Murphy, Gerard Leavey

**Affiliations:** 1 Ulster University, Coleraine, Northern Ireland

## Objective

Over recent decades, there has been a noticeable increase in the prescription of antidepressant medications, particularly among older adults. This study examines the relationship between health outcomes and patterns of antidepressant use among older adults in Northern Ireland (NI).

## Approach

Participants included all (General Practitioner) GP-registered hospital patients aged fifty-five years and above on 01/01/2010 (n =395,872). Administrative data linkage included demographic information (age, sex; urbanicity); data on antidepressant use from the NI Enhanced Prescribing Database (EPD); and hospital patient admissions/discharges. Repeated measures latent class analysis (RMLCA) identified patterns of antidepressant use (2010 to 2020) in relation to the association between these patterns and health-related outcomes in 2021.

## Results

RMLCA identified four latent classes : long-term antidepressant use (17.4%); decreasing antidepressant use (7.1%); increasing antidepressant use (8.4%); no-antidepressant use (67.1%). Those in the long-term and increasing antidepressant use’ classes had the highest average number of hospital visits and recorded chronic conditions in 2021.

## Conclusion

Findings show that two distinct subgroups (comprising 26% of the hospital population) were disproportionately impacted by increased antidepressant prescription rates. Because of their increased likelihood of experiencing higher ill-health, these people are crucial targets for additional psychosocial support.

## Implications

Analyses of this kind emphasize the importance of widening access to administrative data, enabling researchers to identify and examine at-risk groups allowing more targeted policymaking to better address their needs.

Data for this project was provided by the HSC Honest Broker Service; any views or opinions presented are solely those of the author.

